# 4′-Chloro­biphenyl-3-yl 2,2,2-trichloro­ethyl sulfate

**DOI:** 10.1107/S1600536810031338

**Published:** 2010-08-18

**Authors:** Xueshu Li, Sean Parkin, Michael W. Duffel, Larry W. Robertson, Hans-Joachim Lehmler

**Affiliations:** a The University of Iowa, Department of Occupational and Environmental Health, UI Research Campus, 124 IREH, Iowa City, IA 52242-5000, USA; b University of Kentucky, Department of Chemistry, Lexington, KY 40506-0055, USA; c Division of Medicinal and Natural Products Chemistry, College of Pharmacy, University of Iowa, Iowa City, IA 52242, USA

## Abstract

The title compound, C_14_H_10_Cl_4_O_4_S, is a 2,2,2-trichloro­ethyl-protected precursor of 4′-chloro­biphenyl-3-yl sulfate, a sulfuric acid ester of 4′-chloro­biphenyl-3-ol. The C_aromatic_—O and O—S bond lengths of the C_aromatic_—O—S unit are comparable to those in structurally analogous biphenyl-4-yl 2,2,2-trichloro­ethyl sulfates with no electro­negative chlorine substituent in the benzene ring with the sulfate ester group. The dihedral angle between the aromatic rings is 27.47 (6)°.

## Related literature

For similar structures of sulfuric acid biphenyl-4-yl ester 2,2,2-trichloro-ethyl esters, see: Li *et al.* (2008 ▶, 2010*a* ▶,*b* ▶,*c* ▶). For a review of structures of sulfuric acid aryl mono esters, see: Brandao *et al.* (2005 ▶). For further discussion of dihedral angles in chlorinated biphenyl derivatives, see: Lehmler *et al.* (2002 ▶); Shaikh *et al.* (2008 ▶); Vyas *et al.* (2006 ▶). For additional background to hy­droxy­lated polychlorinated biphenyls, see: Bergman *et al.* (1994 ▶); Buckman *et al.* (2006 ▶); Dirtu *et al.* (2010 ▶); Liu *et al.* (2006 ▶, 2009 ▶); Nomiyama *et al.* (2010 ▶); Wang *et al.* (2006 ▶).
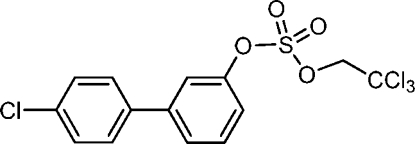


## Experimental

### 

#### Crystal data

C_14_H_10_Cl_4_O_4_S
*M* *_r_* = 416.08
Monoclinic, 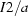

*a* = 21.1900 (3) Å
*b* = 5.8543 (1) Å
*c* = 26.6803 (5) Å
β = 98.304 (1)°
*V* = 3275.06 (10) Å^3^
*Z* = 8
Mo *K*α radiation
μ = 0.87 mm^−1^
*T* = 90 K
0.41 × 0.22 × 0.06 mm

#### Data collection

Nonius KappaCCD diffractometer
Absorption correction: multi-scan (*SCALEPACK*; Otwinowski & Minor, 1997 ▶) *T*_min_ = 0.718, *T*_max_ = 0.950
30021 measured reflections
3759 independent reflections
3242 reflections with *I* > 2σ(*I*)
*R*_int_ = 0.041

#### Refinement

*R*[*F* ^2^ > 2σ(*F* ^2^)] = 0.026
*wR*(*F* ^2^) = 0.067
*S* = 1.05
3759 reflections
208 parameters
H-atom parameters constrained
Δρ_max_ = 0.39 e Å^−3^
Δρ_min_ = −0.38 e Å^−3^

### 

Data collection: *COLLECT* (Nonius, 1998 ▶); cell refinement: *SCALEPACK* (Otwinowski & Minor, 1997 ▶); data reduction: *DENZO-SMN* (Otwinowski & Minor, 1997 ▶); program(s) used to solve structure: *SHELXS97* (Sheldrick, 2008 ▶); program(s) used to refine structure: *SHELXL97* (Sheldrick, 2008 ▶); molecular graphics: *XP* in *SHELXTL* (Sheldrick, 2008 ▶); software used to prepare material for publication: *SHELXL97* and local procedures.

## Supplementary Material

Crystal structure: contains datablocks I, global. DOI: 10.1107/S1600536810031338/om2350sup1.cif

Structure factors: contains datablocks I. DOI: 10.1107/S1600536810031338/om2350Isup2.hkl

Additional supplementary materials:  crystallographic information; 3D view; checkCIF report

## Figures and Tables

**Fig. 1.**
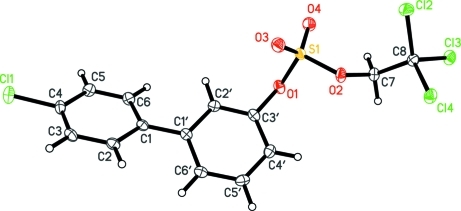
View of the title compound showing the atom-labeling scheme. Displacement ellipsoids are drawn at the 50% probability level.

## supplementary crystallographic information

### Comment

Hydroxylated metabolites of polychlorinated biphenyls (OHPCBs) are present in the serum of humans (Bergman *et al.*, 1994; Dirtu *et al.*, 2010) and other animals (Buckman *et al.*, 2006; Nomiyama *et al.*, 2010). Recent studies also indicate that the mammalian cytosolic sulfotransferases catalyze the formation of sulfuric acid esters from OHPCBs (Liu *et al.*, 2006; Liu *et al.*, 2009; Wang *et al.*, 2006). However, our knowledge of the metabolic and toxicologic significance of these sulfation reactions has been hindered by a lack of information on the structural and chemical properties of the sulfuric acid ester products. As one component of our continuing studies on the properties of the sulfuric acid esters of OHPCBs, we report here the structure of 4'-chloro-biphenyl-3-yl 2,2,2-trichloroethyl sulfate.

Several authors have proposed that the C_aromatic_—O and the corresponding O—S bond lengths are correlated with the stability of sulfuric acid conjugates (Brandao *et al.*, 2005; Li *et al.*, 2010*a*-*c*; Li *et al.*, 2008). The C_aromatic_—O (*i.e.*, C3'-O1) and O—S (*i.e.*, O1—S1) bond lengths of the title compound are 1.4338 (17) Å and 1.5685 (11) Å, respectively. These values are comparable to the corresponding bond lengths reported for analogous biphenyl-4-yl 2,2,2-trichloroethyl sulfates with no electronegative chlorine substituent in the sulfated benzene ring (Li *et al.*, 2010*a*-*c*; Li *et al.*, 2008), which suggest that, analogous to biphenyl-4-yl sulfates, the 4'-chloro-biphenyl-3-yl sulfate corresponding to the title compound may be stable under physiological conditions (Brandao *et al.*, 2005; Li *et al.*, 2010*c*).

The dihedral angle of the biphenyl moiety of OHPCBs and, consequently, their sulfuric acid conjugates is associated with their affinity for cellular target molecules and, therefore, may correlate with their toxicity. The title compound adopts a solid state dihedral angle of 27.47 (6)°. The solid state dihedral angles of structurally related biphenyl-4-yl 2,2,2-trichloroethyl sulfates without *ortho* chlorine substituents ranges from 4.9 (2)° to 41.84 (16)° (Li *et al.*, 2010*a*,*b*; Li *et al.*, 2008). This large range of dihedral angles suggests, similar to the parent PCBs (Lehmler *et al.,* 2002; Shaikh *et al.*, 2008; Vyas *et al.*, 2006), a conformational flexibility of the biphenyl moiety that allows the title compound and analogous biphenyl-4-yl sulfates to adopt an energetically less favorable conformation in the solid state due to crystal packing effects.

### Experimental

The title compound was synthesized from 3',4'-dichlorobiphenyl-4-ol by sulfation with 2,2,2-trichloroethyl sulfonyl chloride using 4-dimethylaminopyridine as catalyst (Li *et al.*, 2010*c*). Crystals suitable for crystal structure analysis were obtained by slowly evaporating a methanolic solution of the title compound.

### Refinement

H atoms were placed in idealized positions and were constrained with distances of 0.99 Å for CH_2_ and 0.95 Å for C_ar_H, and with *U*_iso_(H) = 1.2*U*_eq_ of their attached C atom.

### Crystal data

**Table:**

C_14_H_10_Cl_4_O_4_S	*F*(000) = 1680
*M**_r_* = 416.08	*D*_x_ = 1.688 Mg m^−^^3^
Monoclinic, *I*2/*a*	Mo *K*α radiation, λ = 0.71073 Å
Hall symbol: -I 2ya	Cell parameters from 4127 reflections
*a* = 21.1900 (3) Å	θ = 1.0–27.5°
*b* = 5.8543 (1) Å	µ = 0.87 mm^−^^1^
*c* = 26.6803 (5) Å	*T* = 90 K
β = 98.304 (1)°	Plate, colourless
*V* = 3275.06 (10) Å^3^	0.41 × 0.22 × 0.06 mm
*Z* = 8	

### Data collection

**Table:**

Nonius KappaCCD diffractometer	3759 independent reflections
Radiation source: fine-focus sealed tube	3242 reflections with *I* > 2σ(*I*)
graphite	*R*_int_ = 0.041
Detector resolution: 18 pixels mm^-1^	θ_max_ = 27.5°, θ_min_ = 1.5°
ω scans at fixed χ = 55°	*h* = −27→27
Absorption correction: multi-scan (*SCALEPACK*; Otwinowski & Minor, 1997)	*k* = −7→7
*T*_min_ = 0.718, *T*_max_ = 0.950	*l* = −34→34
30021 measured reflections	

### Refinement

**Table:**

Refinement on *F*^2^	Primary atom site location: structure-invariant direct methods
Least-squares matrix: full	Secondary atom site location: difference Fourier map
*R*[*F*^2^ > 2σ(*F*^2^)] = 0.026	Hydrogen site location: inferred from neighbouring sites
*wR*(*F*^2^) = 0.067	H-atom parameters constrained
*S* = 1.05	*w* = 1/[σ^2^(*F*_o_^2^) + (0.0294*P*)^2^ + 3.9224*P*] where *P* = (*F*_o_^2^ + 2*F*_c_^2^)/3
3759 reflections	(Δ/σ)_max_ = 0.001
208 parameters	Δρ_max_ = 0.39 e Å^−^^3^
0 restraints	Δρ_min_ = −0.38 e Å^−^^3^

### Special details

**Table:**

Geometry. All e.s.d.'s (except the e.s.d. in the dihedral angle between two l.s. planes) are estimated using the full covariance matrix. The cell e.s.d.'s are taken into account individually in the estimation of e.s.d.'s in distances, angles and torsion angles; correlations between e.s.d.'s in cell parameters are only used when they are defined by crystal symmetry. An approximate (isotropic) treatment of cell e.s.d.'s is used for estimating e.s.d.'s involving l.s. planes.
Refinement. Refinement of *F*^2^ against ALL reflections. The weighted *R*-factor *wR* and goodness of fit *S* are based on *F*^2^, conventional *R*-factors *R* are based on *F*, with *F* set to zero for negative *F*^2^. The threshold expression of *F*^2^ > 2σ(*F*^2^) is used only for calculating *R*-factors(gt) *etc*. and is not relevant to the choice of reflections for refinement. *R*-factors based on *F*^2^ are statistically about twice as large as those based on *F*, and *R*- factors based on ALL data will be even larger.

### Fractional atomic coordinates and isotropic or equivalent isotropic displacement parameters (Å^2^)

**Table:**

	*x*	*y*	*z*	*U*_iso_*/*U*_eq_	
S1	0.205499 (17)	0.54226 (7)	0.300571 (14)	0.01597 (9)	
O1	0.18726 (5)	0.4208 (2)	0.24808 (4)	0.0172 (2)	
O2	0.19895 (5)	0.3440 (2)	0.33919 (4)	0.0183 (2)	
O3	0.15849 (5)	0.7045 (2)	0.30865 (4)	0.0221 (2)	
O4	0.27060 (5)	0.6040 (2)	0.30274 (4)	0.0248 (3)	
Cl1	−0.064775 (19)	1.32997 (7)	0.026719 (15)	0.02388 (10)	
Cl2	0.279773 (19)	0.45519 (7)	0.437358 (15)	0.02180 (10)	
Cl3	0.333188 (17)	0.00690 (7)	0.427046 (15)	0.02125 (10)	
Cl4	0.199083 (17)	0.05436 (7)	0.434813 (14)	0.01980 (10)	
C1	0.01131 (7)	0.7573 (3)	0.13260 (5)	0.0141 (3)	
C2	−0.05371 (7)	0.8102 (3)	0.12421 (6)	0.0169 (3)	
H2	−0.0823	0.7255	0.1414	0.020*	
C3	−0.07744 (7)	0.9834 (3)	0.09145 (6)	0.0182 (3)	
H3	−0.1217	1.0176	0.0863	0.022*	
C4	−0.03566 (7)	1.1058 (3)	0.06635 (6)	0.0173 (3)	
C5	0.02901 (7)	1.0572 (3)	0.07324 (6)	0.0179 (3)	
H5	0.0571	1.1412	0.0555	0.021*	
C6	0.05201 (7)	0.8843 (3)	0.10634 (6)	0.0169 (3)	
H6	0.0964	0.8512	0.1114	0.020*	
C1'	0.03627 (7)	0.5740 (3)	0.16854 (5)	0.0139 (3)	
C2'	0.09909 (7)	0.5839 (3)	0.19354 (5)	0.0147 (3)	
H2'	0.1259	0.7090	0.1881	0.018*	
C3'	0.12148 (7)	0.4097 (3)	0.22605 (5)	0.0150 (3)	
C4'	0.08533 (7)	0.2238 (3)	0.23630 (6)	0.0169 (3)	
H4'	0.1025	0.1062	0.2588	0.020*	
C5'	0.02240 (7)	0.2170 (3)	0.21209 (6)	0.0175 (3)	
H5'	−0.0044	0.0935	0.2186	0.021*	
C6'	−0.00152 (7)	0.3877 (3)	0.17880 (5)	0.0155 (3)	
H6'	−0.0444	0.3785	0.1625	0.019*	
C7	0.25272 (7)	0.1927 (3)	0.35354 (6)	0.0166 (3)	
H7A	0.2910	0.2522	0.3406	0.020*	
H7B	0.2431	0.0387	0.3391	0.020*	
C8	0.26470 (7)	0.1800 (3)	0.41106 (6)	0.0153 (3)	

### Atomic displacement parameters (Å^2^)

**Table:**

	*U*^11^	*U*^22^	*U*^33^	*U*^12^	*U*^13^	*U*^23^
S1	0.01324 (17)	0.0176 (2)	0.01656 (19)	−0.00114 (14)	0.00035 (13)	0.00386 (14)
O1	0.0119 (5)	0.0242 (6)	0.0157 (5)	0.0030 (4)	0.0023 (4)	0.0016 (5)
O2	0.0130 (5)	0.0241 (6)	0.0179 (5)	0.0007 (4)	0.0025 (4)	0.0087 (5)
O3	0.0221 (6)	0.0204 (6)	0.0228 (6)	0.0036 (5)	0.0000 (4)	−0.0015 (5)
O4	0.0158 (5)	0.0291 (7)	0.0282 (6)	−0.0073 (5)	−0.0011 (4)	0.0095 (5)
Cl1	0.0251 (2)	0.0248 (2)	0.0220 (2)	0.00861 (17)	0.00412 (15)	0.00689 (16)
Cl2	0.0263 (2)	0.0171 (2)	0.0219 (2)	−0.00104 (15)	0.00333 (15)	−0.00356 (15)
Cl3	0.01544 (17)	0.0210 (2)	0.0267 (2)	0.00532 (15)	0.00114 (14)	0.00543 (16)
Cl4	0.01582 (17)	0.0237 (2)	0.02059 (19)	−0.00115 (15)	0.00512 (14)	0.00656 (15)
C1	0.0139 (7)	0.0161 (7)	0.0124 (7)	−0.0002 (6)	0.0017 (5)	−0.0024 (6)
C2	0.0122 (7)	0.0210 (8)	0.0178 (7)	−0.0024 (6)	0.0032 (5)	−0.0008 (6)
C3	0.0115 (7)	0.0231 (8)	0.0196 (8)	0.0011 (6)	0.0003 (6)	−0.0021 (6)
C4	0.0196 (7)	0.0177 (8)	0.0137 (7)	0.0047 (6)	0.0000 (6)	0.0007 (6)
C5	0.0179 (7)	0.0195 (8)	0.0175 (8)	−0.0004 (6)	0.0066 (6)	0.0017 (6)
C6	0.0120 (7)	0.0203 (8)	0.0190 (7)	0.0020 (6)	0.0048 (5)	−0.0003 (6)
C1'	0.0127 (6)	0.0173 (8)	0.0123 (7)	0.0005 (6)	0.0034 (5)	−0.0017 (6)
C2'	0.0132 (7)	0.0170 (8)	0.0149 (7)	−0.0011 (6)	0.0050 (5)	−0.0005 (6)
C3'	0.0109 (6)	0.0199 (8)	0.0144 (7)	0.0023 (6)	0.0027 (5)	−0.0021 (6)
C4'	0.0213 (7)	0.0158 (8)	0.0143 (7)	0.0018 (6)	0.0051 (6)	0.0006 (6)
C5'	0.0215 (8)	0.0166 (8)	0.0155 (7)	−0.0053 (6)	0.0067 (6)	−0.0028 (6)
C6'	0.0145 (7)	0.0186 (8)	0.0138 (7)	−0.0019 (6)	0.0034 (5)	−0.0033 (6)
C7	0.0168 (7)	0.0180 (8)	0.0152 (7)	0.0029 (6)	0.0030 (5)	0.0024 (6)
C8	0.0136 (7)	0.0145 (7)	0.0180 (7)	0.0016 (6)	0.0031 (5)	0.0009 (6)

### Geometric parameters (Å, °)

**Table:**

S1—O3	1.4153 (12)	C4—C5	1.386 (2)
S1—O4	1.4191 (11)	C5—C6	1.385 (2)
S1—O1	1.5685 (11)	C5—H5	0.9500
S1—O2	1.5714 (11)	C6—H6	0.9500
O1—C3'	1.4338 (17)	C1'—C2'	1.401 (2)
O2—C7	1.4503 (18)	C1'—C6'	1.403 (2)
Cl1—C4	1.7412 (16)	C2'—C3'	1.378 (2)
Cl2—C8	1.7677 (16)	C2'—H2'	0.9500
Cl3—C8	1.7710 (15)	C3'—C4'	1.381 (2)
Cl4—C8	1.7693 (15)	C4'—C5'	1.396 (2)
C1—C2	1.398 (2)	C4'—H4'	0.9500
C1—C6	1.401 (2)	C5'—C6'	1.384 (2)
C1—C1'	1.485 (2)	C5'—H5'	0.9500
C2—C3	1.385 (2)	C6'—H6'	0.9500
C2—H2	0.9500	C7—C8	1.521 (2)
C3—C4	1.385 (2)	C7—H7A	0.9900
C3—H3	0.9500	C7—H7B	0.9900
			
O3—S1—O4	121.62 (8)	C6'—C1'—C1	121.85 (13)
O3—S1—O1	110.62 (6)	C3'—C2'—C1'	119.08 (14)
O4—S1—O1	105.34 (7)	C3'—C2'—H2'	120.5
O3—S1—O2	105.39 (7)	C1'—C2'—H2'	120.5
O4—S1—O2	109.79 (6)	C2'—C3'—C4'	123.88 (14)
O1—S1—O2	102.53 (6)	C2'—C3'—O1	116.77 (13)
C3'—O1—S1	119.08 (9)	C4'—C3'—O1	119.27 (14)
C7—O2—S1	118.95 (9)	C3'—C4'—C5'	116.82 (14)
C2—C1—C6	117.78 (14)	C3'—C4'—H4'	121.6
C2—C1—C1'	120.96 (13)	C5'—C4'—H4'	121.6
C6—C1—C1'	121.26 (13)	C6'—C5'—C4'	120.90 (14)
C3—C2—C1	121.53 (14)	C6'—C5'—H5'	119.6
C3—C2—H2	119.2	C4'—C5'—H5'	119.6
C1—C2—H2	119.2	C5'—C6'—C1'	121.33 (14)
C2—C3—C4	119.01 (14)	C5'—C6'—H6'	119.3
C2—C3—H3	120.5	C1'—C6'—H6'	119.3
C4—C3—H3	120.5	O2—C7—C8	107.85 (12)
C3—C4—C5	121.24 (15)	O2—C7—H7A	110.1
C3—C4—Cl1	119.25 (12)	C8—C7—H7A	110.1
C5—C4—Cl1	119.48 (12)	O2—C7—H7B	110.1
C6—C5—C4	119.01 (14)	C8—C7—H7B	110.1
C6—C5—H5	120.5	H7A—C7—H7B	108.5
C4—C5—H5	120.5	C7—C8—Cl2	110.51 (11)
C5—C6—C1	121.43 (14)	C7—C8—Cl4	110.88 (11)
C5—C6—H6	119.3	Cl2—C8—Cl4	110.07 (8)
C1—C6—H6	119.3	C7—C8—Cl3	106.40 (10)
C2'—C1'—C6'	117.97 (14)	Cl2—C8—Cl3	109.34 (8)
C2'—C1'—C1	120.18 (13)	Cl4—C8—Cl3	109.56 (8)
			
O3—S1—O1—C3'	−24.84 (13)	C2—C1—C1'—C6'	27.7 (2)
O4—S1—O1—C3'	−158.01 (11)	C6—C1—C1'—C6'	−152.88 (15)
O2—S1—O1—C3'	87.12 (11)	C6'—C1'—C2'—C3'	1.4 (2)
O3—S1—O2—C7	−158.44 (11)	C1—C1'—C2'—C3'	−178.90 (13)
O4—S1—O2—C7	−25.84 (13)	C1'—C2'—C3'—C4'	−0.8 (2)
O1—S1—O2—C7	85.75 (11)	C1'—C2'—C3'—O1	176.06 (13)
C6—C1—C2—C3	−0.5 (2)	S1—O1—C3'—C2'	91.88 (14)
C1'—C1—C2—C3	178.90 (14)	S1—O1—C3'—C4'	−91.13 (15)
C1—C2—C3—C4	0.2 (2)	C2'—C3'—C4'—C5'	−0.5 (2)
C2—C3—C4—C5	0.4 (2)	O1—C3'—C4'—C5'	−177.25 (13)
C2—C3—C4—Cl1	−177.88 (12)	C3'—C4'—C5'—C6'	1.2 (2)
C3—C4—C5—C6	−0.7 (2)	C4'—C5'—C6'—C1'	−0.6 (2)
Cl1—C4—C5—C6	177.54 (12)	C2'—C1'—C6'—C5'	−0.7 (2)
C4—C5—C6—C1	0.4 (2)	C1—C1'—C6'—C5'	179.55 (14)
C2—C1—C6—C5	0.2 (2)	S1—O2—C7—C8	129.66 (11)
C1'—C1—C6—C5	−179.25 (14)	O2—C7—C8—Cl2	−58.24 (14)
C2—C1—C1'—C2'	−152.01 (15)	O2—C7—C8—Cl4	64.09 (14)
C6—C1—C1'—C2'	27.4 (2)	O2—C7—C8—Cl3	−176.84 (10)

## References

[bb1] Bergman, Å., Klasson-Wehler, E. & Kuroki, H. (1994). *Environ. Health Perspect.* **102**, 464–469. 10.1289/ehp.94102464 PMC1567124 8593850

[bb2] Brandao, T. A. S., Priebe, J. P., Damasceno, A. S., Bortoluzzi, A. J., Kirby, A. J. & Nome, F. (2005). *J. Mol. Struct.* **734**, 205–209.

[bb3] Buckman, A. H., Wong, C. S., Chow, E. A., Brown, S. B., Solomon, K. R. & Fisk, A. T. (2006). *Aquat. Toxicol.* **78**, 176–185. 10.1016/j.aquatox.2006.02.033 16621064

[bb4] Dirtu, A. C., Jaspers, V. L. B., Cernat, R., Neels, H. & Covaci, A. (2010). *Environ. Sci. Technol.* **44**, 2876–2883. 10.1021/es902149b 20384379

[bb5] Lehmler, H.-J., Parkin, S. & Robertson, L. W. (2002). *Chemosphere*, **46**, 485–488. 10.1016/s0045-6535(01)00177-1 11829405

[bb6] Li, X., Parkin, S., Duffel, M. W., Robertson, L. W. & Lehmler, H.-J. (2010*a*). *Acta Cryst.* E**66**, o1615–o1616. 10.1107/S1600536810020362 PMC3006693 21587847

[bb7] Li, X., Parkin, S., Duffel, M. W., Robertson, L. W. & Lehmler, H.-J. (2010*b*). *Acta Cryst.* E**66**, o1073. 10.1107/S1600536810012845 PMC2979148 21579128

[bb8] Li, X., Parkin, S., Duffel, M. W., Robertson, L. W. & Lehmler, H.-J. (2010*c*). *Environ. Int.* doi:10.1016/j.envint.2009.1002.1005. 10.1016/j.envint.2009.02.005 PMC2939219 19345419

[bb9] Li, X., Parkin, S., Robertson, L. W. & Lehmler, H.-J. (2008). *Acta Cryst.* E**64**, o2464. 10.1107/S1600536808038865 PMC2959825 21581432

[bb10] Liu, Y., Apak, T. I., Lehmler, H.-J., Robertson, L. W. & Duffel, M. W. (2006). *Chem. Res. Toxicol.* **19**, 1420–1425. 10.1021/tx060160+ 17112228

[bb11] Liu, Y., Smart, J. T., Song, Y., Lehmler, H.-J., Robertson, L. W. & Duffel, M. W. (2009). *Drug Metab. Dispos.* **37**, 1065–1072. 10.1124/dmd.108.026021 PMC2677757 19196841

[bb12] Nomiyama, K., Murata, S., Kunisue, T., Yamada, T. K., Mizukawa, H., Takahashi, S. & Tanabe, S. (2010). *Environ. Sci. Technol.* **44**, 3732–3738. 10.1021/es1003928 20426459

[bb13] Nonius (1998). *COLLECT* Nonius BV, Delft, The Netherlands.

[bb14] Otwinowski, Z. & Minor, W. (1997). *Methods in Enzymology*, Vol. 276, *Macromolecular Crystallography*, Part A, edited by C. W. Carter Jr. & R. M. Sweet, pp. 307–326. New York: Academic Press.

[bb15] Shaikh, N. S., Parkin, S., Luthe, G. & Lehmler, H. J. (2008). *Chemosphere*, **70**, 1694–1698. 10.1016/j.chemosphere.2007.07.017 PMC2430941 17723240

[bb16] Sheldrick, G. M. (2008). *Acta Cryst.* A**64**, 112–122. 10.1107/S0108767307043930 18156677

[bb17] Vyas, S. M., Parkin, S. & Lehmler, H.-J. (2006). *Acta Cryst.* E**62**, o2905–o2906.

[bb18] Wang, L.-Q., Lehmler, H.-J., Robertson, L. W. & James, M. O. (2006). *Chem. Biol. Interact.* **159**, 235–246. 10.1016/j.cbi.2005.12.004 16413005

